# Supplementary material for: Cardiovascular Biomarker Profiles in Obesity and Relation to Normalization of Subclinical Cardiac Dysfunction after Bariatric Surgery
Source: Cells. 2022 Jan 26;11(3):422. doi: 10.3390/cells11030422 (PMC8834585; doi:10.3390/cells11030422)
Supplement: Supplementary file 1 [file cells-11-00422-s001.zip › cells-1529973-supplementary.pdf]

Supplementary Table S1: List of abbreviations of all 92 biomarkers

Abbreviation	Full name
**AP-N**	Aminopeptidase N
**AZU**	Azurocidin
**BLM hydrolase**	Bleomycin hydrolase
**CCL15**	C-C motif chemokine 15
**CCL16**	C-C motif chemokine 16
**CCL24**	C-C motif chemokine 24
**CXCL16**	C-X-C motif chemokine 16
**CDH5**	Cadherin-5
**CPA1**	Carboxypeptidase A1
**CPB1**	Carboxypeptidase B
**CASP-3**	Caspase-3
**CTSD**	Cathepsin D
**CTSZ**	Cathepsin Z
**ALCAM**	CD166 antigen
**CHI3L1**	Chitinase-3-like protein 1
**CHIT1**	Chitotriosidase-1
**COL1A1**	Collagen alpha-1(I) chain
**CD93**	Complement component C1q receptor
**CNTN1**	Contactin-1
**CSTB**	Cystatin-B
**SELE**	E-selectin
**PI3**	Elafin
**EPHB4**	Ephrin type-B receptor 4
**EGFR**	Epidermal growth factor receptor
**Ep-CAM**	Epithelial cell adhesion molecule
**FABP4**	Fatty acid-binding protein, adipocyte
**Gal-3**	Galectin-3
**Gal-4**	Galectin-4
**GRN**	Granulins
**GDF-15**	Growth/differentiation factor 15
**IGFBP-1**	Insulin-like growth factor-binding protein 1
**IGFBP-2**	Insulin-like growth factor-binding protein 2
**IGFBP-7**	Insulin-like growth factor-binding protein 7
**ITGB2**	Integrin beta-2
**ICAM-2**	Intercellular adhesion molecule 2
**IL-1RT1**	Interleukin-1 receptor type 1
**IL-1RT2**	Interleukin-1 receptor type 2
**IL-17RA**	Interleukin-17 receptor A
**IL-18BP**	Interleukin-18-binding protein
**IL2-RA**	Interleukin-2 receptor subunit alpha
**IL-6RA**	Interleukin-6 receptor subunit alpha
**JAM-A**	Junctional adhesion molecule A
**KLK6**	Kallikrein-6
**LDL receptor**	Low-density lipoprotein receptor
**LTBR**	Lymphotoxin-beta receptor
**MEPE**	Matrix extracellular phosphoglycoprotein
**MMP-2**	Matrix metalloproteinase-2
**MMP-3**	Matrix metalloproteinase-3
**MMP-9**	Matrix metalloproteinase-9
**TIMP4**	Metalloproteinase inhibitor 4
**MCP-1**	Monocyte chemotactic protein 1
**PRTN3**	Myeloblastin
**MPO**	Myeloperoxidase
**MB**	Myoglobin
**NT-proBNP**	N-terminal prohormone brain natriuretic peptide
**Notch 3**	Neurogenic locus notch homolog protein 3

**OPN**	Osteopontin
**OPG**	Osteoprotegerin
**SELP**	P-selectin
**PON3**	Paraoxonase
**PGLYRP1**	Peptidoglycan recognition protein 1
**PLC**	Perlecan
**PAI**	Plasminogen activator inhibitor 1
**PECAM-1**	Platelet endothelial cell adhesion molecule
**GP6**	Platelet glycoprotein VI
**PDGF subunit A**	Platelet-derived growth factor subunit A
**PCSK9**	Proprotein convertase subtilisin/kexin type 9
**DLK-1**	Protein delta homolog 1
**PSP-D**	Pulmonary surfactant-associated protein D
**RETN**	Resistin
**RARRES2**	Retinoic acid receptor responder protein 2
**CD163**	Scavenger receptor cysteine-rich type 1 protein M130
**SCGB3A2**	Secretoglobin family 3A member 2
**SPON1**	Spondin-1
**ST2**	ST2 protein
**TR-AP**	Tartrate-resistant acid phosphatase type 5
**TFPI**	Tissue factor pathway inhibitor
**t-PA**	Tissue-type plasminogen activator
**TR**	Transferrin receptor protein 1
**TFF3**	Trefoil factor 3
**TLT-2**	Trem-like transcript 2 protein
**TNFSF13B**	Tumor necrosis factor ligand superfamily member 13B
**TNF-R1**	Tumor necrosis factor receptor 1
**TNF-R2**	Tumor necrosis factor receptor 2
**TNFRSF10C**	Tumor necrosis factor receptor superfamily member 10C
**TNFRSF14**	Tumor necrosis factor receptor superfamily member 14
**FAS**	Tumor necrosis factor receptor superfamily member 6
**AXL**	Tyrosine-protein kinase receptor UFO
**SHPS-1**	Tyrosine-protein phosphatase non-receptor type substrate 1
**U-PAR**	Urokinase plasminogen activator surface receptor
**uPA**	Urokinase-type plasminogen activator
**vWF**	von Willebrand factor

Supplementary Table S2: Comparison of all biomarkers pre- and post-bariatric surgery

Abbreviation	Pre-surgery (n=72)	Post-surgery (n=72)	Relative changes	p-value
**AP-N**	36.6 [33.1-41.8]	37.4 [33.3-42.3]	2.2%	0.27
**AZU**	6.1 [5.0-8.0]	5.3 [4.6-6.5]	-13.1%	**<0.001***
**BLM hydrolase**	8.0 [6.7-9.5]	6.7 [5.4-7.8]	-16.3%	**<0.001***
**CCL15**	117 [103-154]	135 [115-172]	15.3%	0.75
**CCL16**	126 [91-158]	119 [92-147]	-5.6%	**<0.001***
**CCL24**	40.9 [24.4-67.5]	40.8 [26.4-69.0]	-0.2%	0.75
**CXCL16**	48.2 [43.2-55.9]	43.0 [38.8-49.9]	-10.8%	**<0.001***
**CDH5**	26.1 [22.0-29.7]	21.8 [18.9-25.9]	-16.5%	**<0.001***
**CPA1**	74.1 [51.3-96.8]	70.7 [53.3-102.6]	-4.6%	0.92
**CPB1**	65.5 [45.4-87.2]	63.2 [49.0-89.8]	-3.5%	0.26
**CASP-3**	529 [335-751]	336 [198-532]	-36.5%	**<0.001***
**CTSD**	6.5 [5.5-8.4]	5.5 [4.7-6.7]	-15.4%	**<0.001***
**CTSZ**	50.9 [42.6-63.5]	47.2 [42.6-53.5]	-7.3%	**<0.001***
**ALCAM**	224 [205-251]	207 [181-228]	-7.6%	**<0.001***
**CHI3L1**	17.3 [11.9-26.1]	18.0 [11.3-26.9]	4.0%	0.49
**CHIT1**	29.7 [19.4-40.3]	28.8 [19.3-44.8]	-3.0%	0.15
**COL1A1**	8.6 [7.2-10.1]	11.7 [9.8-13.3]	36.0%	**<0.001***
**CD93**	2349 [2074-2722]	2239 [1842-2415]	-4.7%	**0.001***
**CNTN1**	29.3 [24.9-34.3]	31.6 [26.1-36.1]	7.8%	**<0.001***
**CSTB**	21.1 [16.1-28.6]	18.3 [14.1-21.2]	-13.3%	**<0.001***
**SELE**	6035 [4054-7535]	3604 [2477-4478]	-40.3%	**<0.001***
**PI3**	5.4 [4.5-7.3]	5.3 [4.2-7.0]	-1.9%	**0.019***
**EPHB4**	51.9 [47.4-59.9]	52.5 [47.2-59.8]	1.2%	0.22
**EGFR**	11.3 [10.5-12.5]	10.0 [9.1-10.7]	-11.5%	**<0.001***
**Ep-CAM**	56.6 [28.4-94.1]	107.3 [58.5-204.4]	89.6%	**<0.001***
**FABP4**	98.3 [60.9-135.9]	68.8 [47.9-85.2]	-30.0%	**<0.001***
**Gal-3**	11.5 [10.4-12.9]	11.2 [10.1-12.6]	-2.6%	0.35
**Gal-4**	15.6 [12.7-19.8]	19.6 [14.9-25.9]	25.6%	**0.002***
**GRN**	57.1 [49.8-66.1]	57.2 [47.5-63.8]	0.2%	**0.003***
**GDF-15**	47.6 [38.5-59.7]	51.8 [40.1-63.1]	8.8%	**0.048**
**IGFBP-1**	13.4 [6.6-23.9]	36.8 [20.8-52.4]	174.6%	**<0.001***
**IGFBP-2**	450 [338-641]	188 [136-286]	-58.2%	**<0.001***
**IGFBP-7**	270 [236-304]	252 [222-294]	-6.7%	0.09
**ITGB2**	188 [136-286]	450 [337-641]	139.4%	**<0.001***
**ICAM-2**	53.8 [44.8-61.3]	58.1 [48.3-68.4]	9.0%	**<0.001***
**IL-1RT1**	93.2 [79.8-105.7]	96.3 [88.7-109.8]	3.3%	**<0.001***
**IL-1RT2**	48.6 [40.8-54.5]	40.3 [33.3-46.6]	-17.1%	**<0.001***
**IL-17RA**	22.1 [18.1-28.9]	19.6 [16.3-25.2]	-11.3%	**<0.001***
**IL-18BP**	71.1 [63.9-81.4]	70.4 [60.5-79.9]	-1.0%	**0.007***
**IL2-RA**	15.0 [11.9-17.7]	13.3 [11.8-17.2]	-11.3%	**<0.001***
**IL-6RA**	5075 [4208-6023]	4850 [4003-5553]	-4.4%	**<0.001***
**JAM-A**	110.2 [74.7-161.9]	84.0 [51.3-127.5]	-23.8%	**<0.001***
**KLK6**	5.9 [5.1-6.8]	6.2 [5.2-6.9]	5.1%	**<0.001***
**LDL receptor**	25.4 [19.5-32.9]	14.9 [11.6-19.8]	-41.3%	**<0.001***
**LTBR**	17.0 [15.3-19.2]	17.0 [14.8-18.8]	0%	0.35
**MEPE**	71.2 [60.3-88.7]	73.4 [62.3-90.2]	3.1%	**0.025***
**MMP-2**	17.4 [14.9-19.5]	18.8 [16.3-22.4]	8.0%	**<0.001***
**MMP-3**	194 [154-278]	177 [132-234]	-8.8%	0.22
**MMP-9**	58.8 [40.7-89.8]	49.2 [33.2-61.6	-16.3%	**<0.001***
**TIMP4**	13.2 [11.4-15.2]	14.2 [11.8-17.0]	7.6%	**0.047**
**MCP-1**	17.4 [14.8-21.1]	16.2 [13.3-18.9]	-6.9%	**<0.001***
**PRTN3**	18.2 [14.5-21.6]	16.3 [13.8-18.9]	-10.4%	**<0.001***
**MPO**	11.2 [9.5-13.5]	10.0 [8.6-12.4]	-10.7%	**<0.001***
**MB**	212 [170-292]	163 [130-213]	-23.1%	**0.003***

**NT-proBNP**	11.1 [6.6-16.6]	17.5 [11.2-24.6]	57.7%	**0.010***
**Notch 3**	50.2 [41.2-59.1]	52.6 [42.5-63.3]	4.9%	**0.021***
**OPN**	198 [157-248]	315 [257-400]	59.1%	**<0.001***
**OPG**	16.7 [13.6-18.35]	15.7 [14.1-18.3]	-6.0%	0.12
**SELP**	2615 [1877-3616]	1841 [1210-2617]	-29.6%	**<0.001***
**PON3**	107 [77-156]	139 [106-159]	29.9%	**<0.001***
**PGLYRP1**	188 [154-228]	178 [148-204]	-5.3%	**<0.001***
**PLC**	299 [266-337]	284 [249-309]	-5.0%	**<0.001***
**PAI**	77.6 [53.7-109.1]	44.5 [31.0-64.9]	-42.7%	**<0.001***
**PECAM-1**	70.4 [52.6-92.3]	54.8 [39.6-75.0]	-22.2%	**<0.001***
**GP6**	17.4 [11.5-23.2]	13.2 [8.7-18.4]	-24.1%	**<0.001***
**PDGF subunit A**	25.2 [18.7-36.1]	17.6 [13.2-25.9]	-30.2%	**<0.001***
**PCSK9**	8.9 [7.4-10.5]	7.7 [7.0-8.8]	-13.5%	**<0.001***
**DLK-1**	53.8 [36.5-66.9]	78.7 [57.7-97.1]	45.3%	**<0.001***
**PSP-D**	7.0 [5.4-10.3]	6.5 [4.5-8.1]	-7.1%	**0.007***
**RETN**	82.0 [68.5-100.8]	83.0 [62.6-97.2]	1.2%	**0.037***
**RARRES2**	4461 [3888-4950]	3489 [3100-4032]	-21.8%	**<0.001***
**CD163**	299.4 [249.6-389.6]	220.5 [175.9-301.6]	-26.4%	**<0.001***
**SCGB3A2**	5.4 [4.1-7.8]	5.9 [4.8-8.7]	9.3%	**0.009***
**SPON1**	4.5 [4.1-5.1]	4.9 [4.2-5.5]	8.9%	**0.024***
**ST2**	23.3 [18.3-29.3]	24.2 [18.1-30.3]	3.9%	0.45
**TR-AP**	15.3 [13.2-17.7]	14.7 [12.8-16.8]	-3.9%	**0.009***
**TFPI**	736 [654-847]	674 [578-780]	-12.1%	**<0.001***
**t-PA**	111 [84-155]	74 [61-100]	-33.3%	**<0.001***
**TR**	59.5 [44.1-83.9]	45.2 [37.8-56.6]	-24.0%	**<0.001***
**TFF3**	36.9 [31.7-46.0]	43.5 [38.0-55.0]	17.9%	0.67
**TLT-2**	61.9 [52.7-73.6]	52.8 [43.8-62.3]	-14.7%	**<0.001***
**TNFSF13B**	168 [142-189]	142 [121-159]	-15.5%	**<0.001***
**TNF-R1**	133 [115-150]	122 [101-138]	-8.3%	**<0.001***
**TNF-R2**	66.7 [55.3-77.8]	61.0 [52.1-70.4]	-8.5%	**<0.001***
**TNFRSF10C**	155 [122-202]	155 [103-203]	0%	**<0.001***
**TNFRSF14**	36.1 [30.7-42.1]	34.0 [29.8-39.4]	-5.8%	**<0.001***
**FAS**	76.4 [67.4-87.2]	71.5 [62.0-81.7]	-6.4%	**0.0013***
**AXL**	637 [553-726]	588 [507-676]	-7.7%	**0.012***
**SHPS-1**	15.0 [12.9-18.4]	15.6 [12.9-18.0]	4.0%	0.51
**U-PAR**	48.8 [40.9-60.1]	45.8 [40.5-52.7]	-6.1%	**<0.001***
**uPA**	29.9 [26.2-34.7]	26.6 [21.8-31.0]	-11.0%	**<0.001***
**vWF**	153 [108-216]	149 [122-196]	-2.6%	0.07

Values represent median [Q1-Q3], all units are NPX.

Relative changes of all biomarkers from pre- to one year post-bariatric surgery were calculated by subtracting the median value of biomarkers pre-surgery from the value of the biomarkers post-surgery, and dividing the obtained difference by the median value of the biomarkers pre-surgery.

P-values displayed for changes in biomarker levels between pre- and post-surgery were obtained by univariable linear mixed modeling.

* Significant after Benjamini–Hochberg correction.

Supplementary Table S3: Biomarkers selected by spls-DA analysis

TLT2
GP6
CASP3
SELP
JAMA
PECAM1
PDGFsubunitA
